# A deep learning model identifies emphasis on hard work as an important predictor of income inequality

**DOI:** 10.1038/s41598-022-13902-x

**Published:** 2022-06-14

**Authors:** Abhishek Sheetal, Srinwanti H. Chaudhury, Krishna Savani

**Affiliations:** 1 School of Business and Law, Central Queensland University, Rockhampton, Australia; 2 Faculty of Business, The Hong Kong Polytechnic University, Kowloon, Hong Kong; 3 Business School, University of Queensland, Brisbane, Australia; 4 Nanyang Business School, Nanyang Technological University, Singapore, Singapore

**Keywords:** Psychology, Human behaviour

## Abstract

High levels of income inequality can persist in society only if people accept the inequality as justified. To identify psychological predictors of people’s tendency to justify inequality, we retrained a pre-existing deep learning model to predict the extent to which World Values Survey respondents believed that income inequality is necessary. A feature importance analysis revealed multiple items associated with the importance of hard work as top predictors. As an emphasis on hard work is a key component of the Protestant Work Ethic, we formulated the hypothesis that the PWE increases acceptance of inequality. A correlational study found that the more people endorsed PWE, the less disturbed they were about factual statistics about wealth equality in the US. Two experiments found that exposing people to PWE items decreased their disturbance with income inequality. The findings indicate that machine learning models can be reused to generate viable hypotheses.

In recent decades, levels of income inequality have increased sharply in most countries around the world, particularly in economically developed countries^1^. In fact, the income gap between the rich and poor has been increasing exponentially over the past half-century^2,3^. For instance, in the United States, the three richest men own more wealth than the bottom 50% of Americans combined^4^. Globally, the 26 wealthiest people own as much wealth as the 3.8 billion poorest people (i.e., about half the world's population)^5^. The ongoing COVID-19 pandemic has further exacerbated income inequality, as many low-wage earners lost their jobs, but most high-wage earners retained their jobs^6^.

At the national level, high degrees of income inequality has also been associated with a number of negative psychological outcomes, including lower trust^7^, lower subjective well-being^8^, lower life satisfaction^9^, and worse self-reported health^10–13^. For example, less well-off people experience more negative affect because of their chronic economic vulnerability^14,15^. Moreover, even wealthy individuals experience more distress and anxiety in societies with high degrees of inequality because they can always compare themselves with others who are even wealthier^16^.

Recent research on inequality in psychology has found that although people have little idea about the extent of income inequality prevalent in most societies, individuals across the political spectrum prefer a more equal income distribution^17^. However, another study found that a majority of people preferred an unequal income distribution in which the richest quintile earned 50 times more than the poorest quintile^18^. Nevertheless, both studies found substantial individual differences—some people think that a high degree of income inequality is justified, whereas others think it is unjust. What are the psychological predictors that shape whether people desire equality or inequality? Many individuals might be motivated to rationalize income inequality even if they are disadvantaged by the inequality because of their psychological need to perceive the social system as fair and just^19^. Further, the more people think that they have a chance to move up the income ladder, the less concerned they are about high degrees of income inequality^20,21^. Moreover, the greater the salience of choice, the less concerned people are about high degrees of income inequality because they attribute rich people's outcomes to their good choices and poor people's outcomes to their bad choices^22,23^.

Despite the volume of research on predictors of people’s concern for income inequality, it is unclear what are the most important predictors. Indeed, this issue is not specific to this literature—social scientists typically focus on identifying individual predictors of an outcome one at a time (e.g., “Does A cause Z?” “Does B cause Z?” … “Does Y cause Z controlling for A and F?”), not on identifying the most important predictors of the outcome (“Of A, B, C, … Y, which are the most important predictors of Z?”). Answering the latter questions requires engaging in abductive reasoning rather than deductive reasoning, the predominant form of reasoning in the social sciences^24–26^.

Given the importance of income inequality in shaping a wide range of societal and psychological outcomes^1,3,11–13^, we sought to uncover the most important psychological constructs underlying people’s attitudes toward income inequality from a wide range of constructs measured in the World Values Survey^27^. Instead of basing our investigation on the literature or intuition, we used a machine learning method to identify the most important predictors of people's attitudes toward income inequality from hundreds of potential predictors^28^. Natural scientists have argued that “human activities [are] a principal bottleneck in scientific progress” that make “scientific advancement more subject to error and harder to reproduce”^29,30^. Artificial intelligence systems provide an alternate means for generating research ideas free from the limits of human ideation and conceptualization. In a recent article, researchers^31^ used a deep learning model to generate a hypothesis about the predictors of unethical behavior. Their model identified optimism about the future of humanity as one of the top predictors of ethicality. Despite extensive research on both optimism and ethicality, past research has not connected the two. Subsequent studies by the researchers^31^ verified that increasing people's optimism increased their willingness to engage in unethical behavior.

This work demonstrates that instead of generating hypotheses based on their intuition or the literature, social scientists can use machine learning methods to generate hypotheses. Social science researchers often conduct qualitative studies (e.g., ethnographic observations, interviews, and focus groups) to generate hypotheses and to inform existing theories^32^; machine learning can play a similar role but it is more systematic and replicable than qualitative methods. Although machine learning is a theory-blind method for generating hypotheses, researchers can situate the resulting hypotheses in the existing literature. If hypotheses generated by the machine learning model already feature in the literature, then research can either confirm or contradict existing theories. If the hypotheses generated cannot be derived from the existing literature, then researchers can use the hypotheses as the basis for new theorizing. Indeed, scholars agree that social science theories are imprecise and incomplete^33^, but most researchers still focus on hypotheses that can be derived from existing theories. Machine learning methods present a way to potentially move beyond the constraints of existing theories.

When conducting exploratory analyses with large datasets, such as the World Values Survey, ad hoc data mining can yield hundreds of statistically significant bivariate correlations. However, several important relationships might be nonlinear in nature and thus would not be picked up by linear correlations. Alternatively, variables that are weakly correlated or uncorrelated with the outcome variable might still play an important role in predicting the outcome variable through any number of interactions. Machine learning methods avoid these pitfalls by modeling any number of nonlinear relationships and complex interactions. Additionally, when permutation-based feature importance analyses^27^ are applied to machine learning methods, they rank order all predictor variables in terms of their total impact on the outcome variable. Thus, using machine learning methods to identify the most important predictors of an outcome variable of interest provides behavioral scientists with a “principled methodology for working with large datasets”^34^.

In the current research, we chose to undertake a similar theory-blind data-driven approach^31^ to uncover the most important predictors of people’s tendency to justify income inequality. The model might either identify predictors that have already been studied in the literature or identify novel predictors. In either case, it is useful for social scientistic to know which of hundreds of potential predictions are most relevant to people’s attitudes about income inequality.

Notably, the process of building a machine learning model from scratch is time consuming. This is particularly the case for deep neural networks, which have many free parameters that need to be fixed based on trial and error, and other parameters that need to be tuned based on a hyperparameter search procedure^35^. We propose that researchers do not need to build deep neural networks from scratch; instead, they can reuse existing models built on the same dataset if such models are available, and simply retrain the pre-existing model to predict their outcome variable of interest. Such a procedure can substantially reduce the model building time and allow a wider range of researchers to use machine learning methods to generate hypotheses.

## Study 1: Machine Learning

The goal of Study 1 was to identify the most important predictors of the extent to which people justify income inequality from the wide range of attitudes, values, and beliefs measured in the World Values Survey (WVS)^27^, which has been used in recent research to generate hypotheses using machine learning^31^. The version of WVS that we used contained data from people from 98 countries collected over six waves (from 1981–1984 to 2010–2014). Therefore, individuals’ responses are correlated within countries and within wave. The nested structure of the data poses a challenge to regression-based methods, which assume independently distributed errors. Researchers using regression-based methods need to employ hierarchical modelling because the errors of individuals within a given country or sampled in a given wave are likely correlated. However, machine learning models make no such assumptions, and so there is no need for hierarchical modelling. Indeed, explicitly enforcing hierarchical structure in deep learning has shown no discernible benefit^34^. Additionally, our goal was to identify predictors of people’s attitudes about inequality that generalize beyond time and country. By explicitly removing the concept of time and country from the dataset, the model provided us with a hypothesis that could potentially be valid in a wide range of contexts.

## Method

For our dependent measure, we used variable e035 in the WVS dataset, which asked people to indicate their belief about income inequality on a bipolar scale ranging from *1: Income should be made more equal* to *10: We need larger income differences as incentives*. We used the imputed dataset from our previously published paper^31^ for this analysis. Our method was virtually identical to that used in the previous paper^31^. A key difference was that instead of building a deep learning model that classifies respondents into one of two categories (e.g., ethical vs. unethical), we built a deep learning model that predicts respondents' attitudes about income inequality on a 1 to 10 scale.

**Figure 1:**
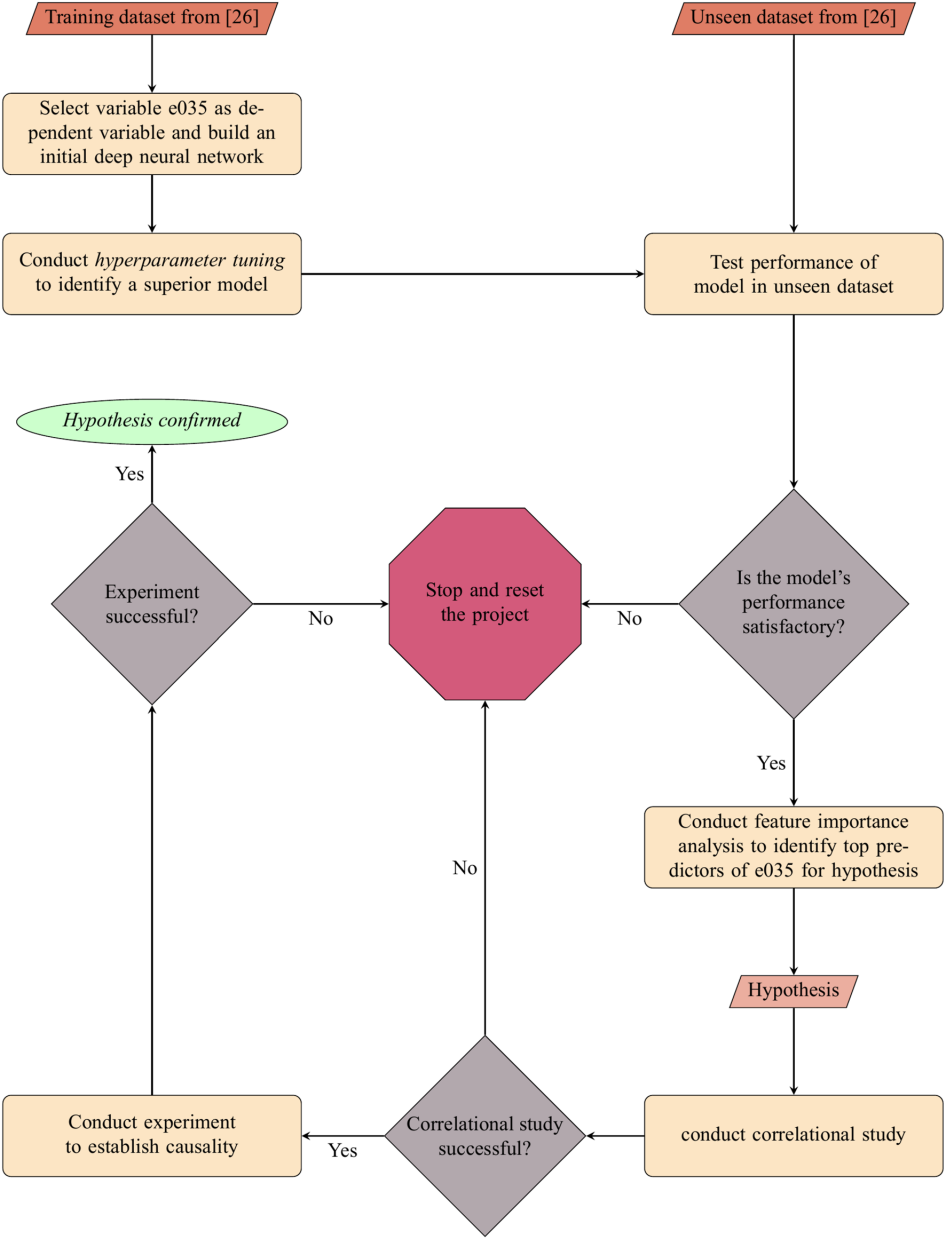
Illustration of the project procedure.

As our goal was to identify psychological predictors of attitudes about income inequality, we excluded all demographic variables assessed in the WVS. We used the same project configuration files used in the previously published research^31^. Figure 1 illustrates the study procedure. The full list of variables that were either excluded or one-hot coded (i.e., a new variable was created for each categorical response option) is provided on the OSF page for this project: https://osf.io/q5xzy. We had 568 variables in the final data file. Of the 336,306 respondents in the dataset in our previous project^26^, 302,732 were included in the *seen* data, and 33,574 in the *unseen* data.

As this model used the same WVS data as in previous research^31^, we used the same model parameters, including the number of layers (see Table 1). However, we retrained all the weights so that the model is learning to predict individuals’ support for inequality based on all the predictor variables. A model using the parameter values from past research using the same dataset but a different outcome variable is unlikely to be the ideal model—if we redid the hyperparameter search process, we would probably identify a model with higher accuracy. However, doing so involves costs in terms of time and computing power. If our goal was to maximize the model's predictive power, then redoing the hyperparameter search would have been essential. However, our focus was on the top predictors of people’s support for income inequality. Although a model with different parameters is likely to have higher accuracy, it is unlikely that the top predictors would change much. We thus decided to skip the hyperparameter search process. More generally, as long as other researchers are using the dataset from past research and are interested in identify the top predictors rather than maximizing predictive accuracy, they can use the relevant model from the previous research and just retrain all the weights^36^.

**Table 1:** Parameters of the final deep learning model, and parameter range.

Parameter	Value	Range
Nodes in 1st layer	900	800–900
Nodes in 2nd layer	479	100–500
Nodes in 3rd layer	225	100–500
Nodes in 4th layer	46	10–50
Dropped connection rate for 1st layer	0.2101	0.1–0.9
Dropped connection rate for 2nd layer	0.166	0.1–0.9
Dropped connection rate for 3rd layer	0.6732	0.1–0.9
Dropped connection rate for 4th layer	0.1455	0.1–0.9
Learning rate	460 × E^−^^10^	[10–500] × E^−^^10^
Batch size	64	64, 128
Kernel initializer	He-uniform	–
Activation function in 1st three layers	ReLU	–
Activation function in output layer	Linear	–
Optimizer	Adam	–
Maximum Epochs	200	–
Learning rate patience	5	–
Early stopping patience	10	–

After the deep learning model was trained using the *seen* data, we presented the model with the *unseen* data and asked it to predict these participants’ attitudes about income inequality. Thus, the participants in the *unseen* data served as “new participants” that the model was never exposed to. Notably, the three questions related to hard work were dispersed all over the WVS and not asked in proximity to the income inequality question.

## Results

**Figure 2:**
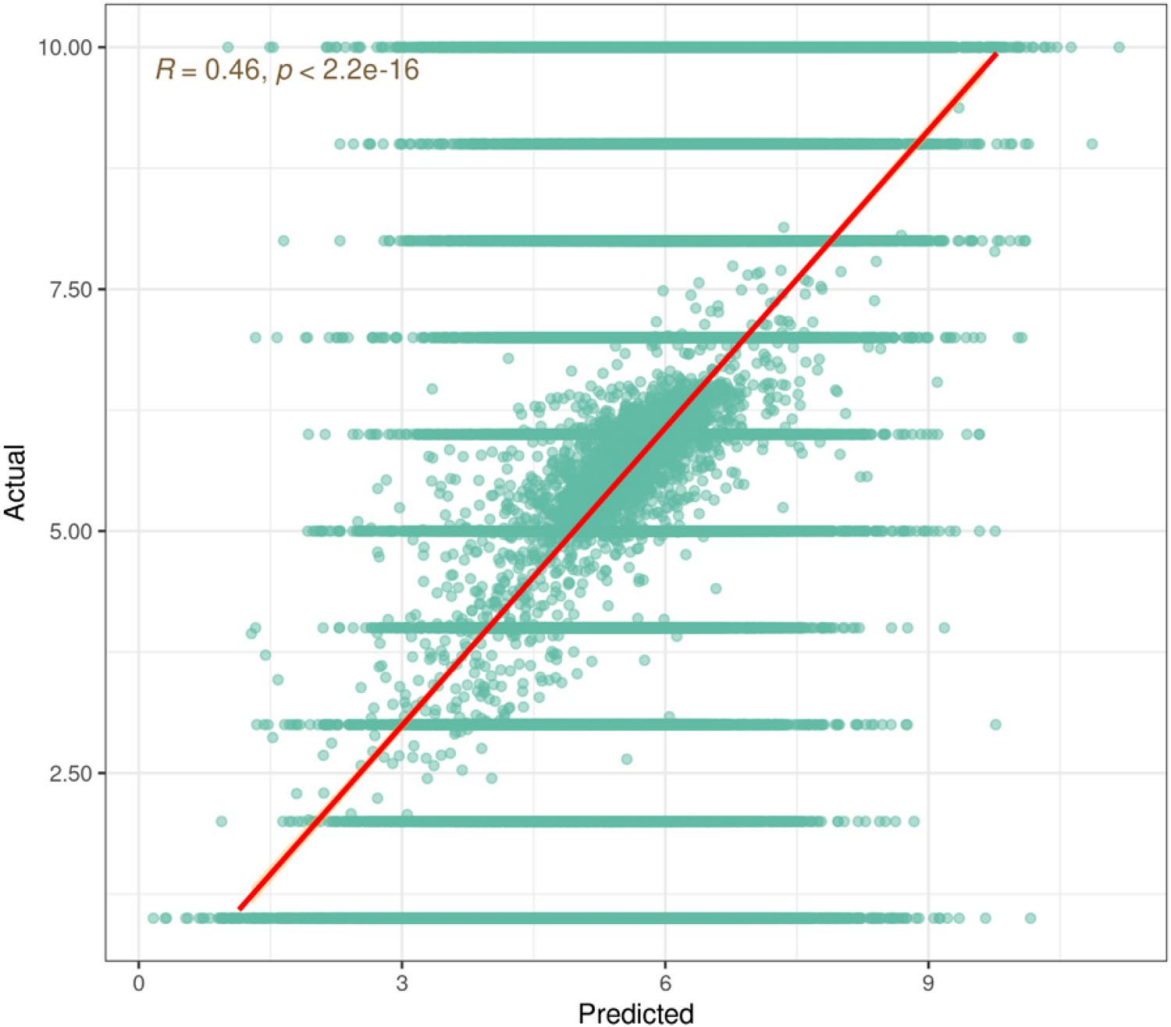
The model’s predicted versus participants’ actual attitudes about inequality in the unseen data.

In the *unseen* data, there was a significant correlation between the model’s predicted attitude about income inequality and participants’ actual attitude about income inequality, Spearman ρ = 0.46, *p* < 2.2 e^−16^ (see Fig. 2). We next conducted a feature importance analysis using the DALEX package^37^ in the *seen* data. This package shuffled the values of each predictor variable one at a time and assessed the change in the model's mean square error. The greater the change is, the greater the importance of the variable in the model as a whole. Note that this procedure ranks variables in terms of their total contribution to the model, including linear and non-linear effects, and main effects and interactions. In other words, the variables are identified and ranked in order of their predictive importance to the dependent variable of income inequality acceptance. The top 10 predictors according to this feature importance analysis are depicted in Table 2.

**Table 2:** Top 10 predictors of acceptance of income inequality based on the feature importance analysis.

WVS variable	Item	Dropout loss
e037	Whether the government or people should take more responsibility to provide for themselves	2.5470
e041	Whether wealth accumulation is win-win or win-lose	2.4730
e036	Whether private ownership or government ownership of business should be increased	2.4703
e033	Self-positioning in political scale (left vs. right)	2.4645
e039	Whether competition is good or harmful	2.4632
a030	Important child qualities: hard work	2.4599
e015	Whether less importance placed on work in the future would be a good thing or a bad thing	2.4588
f053	Believe in hell	2.4586
e040	Hard work brings success	2.4584
f034 (response option 1)	I am a religious person	2.4582

*Dropout loss* refers to the change in the model’s mean square error when the values of the relevant variable are shuffled.

The first five predictors of people’s attitudes about income inequality all assessed participants’ attitudes and beliefs about politics and the economy, and appear closely associated with political orientation. These predictors are consistent with past research^19^ arguing that the justification of inequality is a key component of political orientation. Additionally, these questions were asked in close proximity to the item about income inequality (variable e035) that serves as our dependent measure, so thematic overlap could provide one explanation for why these variables showed up as top predictors. Of the next five predictors, two tapped religiosity, consistent with past research showing that income inequality increases religiosity^38,39^. The remaining three items were related to the importance of hard work: whether hard work is an important quality in children (variable e030), whether hard work leads to success (variable e040), and whether less importance on work would be a good thing (variable e015). An examination of the partial dependence plots (see Fig. S1 in Supplementary Materials) indicated that a greater emphasis on hard work was associated with a greater justification of income inequality.

Hard work takes different meanings in different countries. The Protestant work ethic (PWE)^40^ argues that hard work reflects moral virtue and is a key determinant of success in life^41,42^. Sociologist Max Weber^43,44^ argued that the Protestant work ethic played an important role in fueling capitalism in Western Europe and North America because it allows people to believe that individuals who succeeded due to their hard work are also morally virtuous^40^. Importantly, although the Protestant work ethic is rooted in Calvinist philosophy^45^, it is endorsed by people from many other cultures. Indeed, one study found that Taiwanese employees endorsed PWE more than American and British employees^46^. Scholars have argued that there is a parallel concept of Confucian work ethic in East Asian cultures^47,48^, and of Islamic work ethic in West Asia^49^. Thus, the construct measured in the WVS—importance of hard work—is likely meaningful in a wide range of cultures and is embedded in different culture-specific ideologies.

Although the machine learning model identified culture-general predictors of people’s attitudes about inequality, we decided to conduct our follow-up studies in the US because we had easy access to high quality respondents from the US at a reasonable cost. The Protestant work ethic serves as a foundational principle in in the US^50^, and seems to be the closest cultural construct to the WVS items about hard work, which were identified among the 10 most important predictors of people’s support for inequality. Hence, in the remainder of this research, we focused our attention on the relationship between PWE and support for income inequality in the US.

### Protestant work ethic and attitudes about income inequality

The PWE has numerous psychological and behavioral consequences. For example, at the country level, Protestant values are thought to motivate hard work and long working hours^51^, which can lead to higher per capita income and faster GDP growth^52^. At the individual level, a stronger belief in PWE is associated with higher job satisfaction^53^, greater motivation to spend time even on repetitive, monotonous tasks^54^, and lower psychological well-being following unemployment^50^.

Despite its many positive consequences, PWE has also been theorized as a source of prejudice against disadvantaged groups^55^. Specifically, researchers found that the more people endorsed PWE, the more negative their attitudes toward the poor^56^, African Americans^41^, and obese individuals^42^. The underlying explanation is that PWE emphasizes personal responsibility for outcomes. So when people who endorse PWE consider others who have encountered negative outcomes, they attribute negative outcomes to personal rather than situational factors^55^. Nevertheless, some evidence suggests that PWE might not directly cause negative attitudes toward disadvantaged groups. For example, some researchers^57^ found that among European American children and adolescents, PWE was associated with greater egalitarianism and *less* prejudice toward African Americans. However, in the same study, PWE was associated with weaker egalitarianism and greater prejudice among European American adults. The authors argued that while the definition of PWE is more egalitarian in nature, people in the US often use PWE to justify prejudice^57^.

Past research documenting the link between PWE and racism, sexism, and other forms of biases (e.g., against obese people) is consistent with the machine learning model’s predictions that PWE is associated with greater support for inequality. Whereas past research on PWE has focused on people’s attitudes toward disadvantaged groups, income inequality arises from the distribution of resources across low, middle, and high-income groups. In particular, the high degree of inequality present in many present-day societies is defined by a concentration of wealth at the top end of the income distribution^1,3^. Even if PWE leads to less sympathy for poor people, it does not necessarily imply that PWE makes people less concerned about the concentration of wealth. We thus submit that the hypothesis generated by the machine learning model complements existing research on the link between PWE and prejudice.

We conducted a correlational study and an experiment to verify whether the PWE is a cause of the extent to which people accept income inequality. In both studies, we report all participants, conditions, and measures. We do not have a "file drawer" of unreported studies—these were the only two studies conducted for this project. The verbatim survey materials, data, and analysis code are available on https://osf.io/q5xzy/?view_only=0f18c84a426a4fb8bbb8f40b42b9a4d6.

### Study 2: Correlational study

The goal of Study 2 was to replicate the machine learning model’s finding as well as prior correlational evidence supporting links of religion and economic attitudes^52^ that higher PWE is associated with a greater justification of income inequality using established psychological measures.

## Method

As this was the first study in this project, we did not have an effect size from a prior study to use as a basis for conducting a power analysis. We decided on a sample size of 200, which would give us 80% power to detect a correlation coefficient of *r* = 0.20 with a = 0.05 (two-tailed). A survey seeking 200 US residents was posted on Cloud Research (formerly TurkPrime). We used Cloud Research's in-built feature for excluding low-quality participants and only allowed participants who had completed at least 500 HITs and had an approval rating of 97% or higher. Participants were only allowed to proceed to the survey if they correctly answered four multiple-choice attention-check questions. In response, 200 participants completed our survey. As our focus was on inequality within the US context, we recruited 187 participants who indicated that they were born in the US (96 women, 89 men, 2 other gender; mean age 42.24 years, *SD* = 12.95).

We measured PWE beliefs using the scale developed by past research^41^ (p. 905; sample item: “Anyone who is willing and able to work hard has a good chance of succeeding”). Of the 11 items included in the scale, the final item got inadvertently left out. Participants were asked to respond on a 7-point scale ranging from *strongly disagree* to *strongly agree* (α = 0.83). Next, we adapted a measure of concern about income inequality^22^. Specifically, we presented participants with five factual statistics about existing income and wealth inequality in the US (e.g., "Recent statistics show that 3 richest men in the US own more wealth than the bottom 50% of Americans *combined*"; see Supplementary Materials for all inequality items). For each of the items, participants were asked, "How disturbed are you by this finding?" on a scale ranging from "0 (Not very disturbed)" to "100 (Extremely disturbed)" (α = 0.96). Finally, as both PWE and beliefs about inequality are associated with political orientation^19,45^, we assessed participants' political orientation using a three-item 7-point semantic-differential measure (strongly liberal—strongly conservative, strongly left—strongly right, strongly Democrat—strongly Republican; α = 0.96).

## Results

Preliminary analyses indicated that the dependent variable was highly right-skewed (*M* = 69.24, *SD* = 30.30, skewness =  − 0.98, kurtosis = 2.83; See Fig. S2 in Supplementary Materials for the histogram), and non-normally distributed (skewness-kurtosis test c^2^(*df* = 2) = 19.23, *p* < 0.001. We thus analyzed the data using the nonparametric Spearman's rank-order correlation. We found that the more participants endorsed PWE, the less disturbed they were by information about income inequality in the US, ρ(*N* = 187) =  − 0.35, *p* < 0.001. In a secondary analysis, we computed the partial correlation after accounting for political orientation. As expected, more conservative participants were less disturbed by information about income inequality, ρ = − 0.36, *p* < 0.001. However, participants who endorsed PWE more were also less disturbed, ρ = − 0.17, *p* = 0.022.

Study 2 thus verified the finding from the machine learning model: the more people endorse PWE, the less disturbed they are by factual information about extreme levels of income inequality in the US.

### Study 3: Experiment

The goal of Study 3 was to assess whether PWE causally increases acceptance of income inequality. As PWE is a continuum, we assigned participants to either a high PWE or a low PWE condition. We employed a biased questionnaire manipulation^58^ to assess the causal effect of PWE.

## Method

As this was our first experiment in this project, we did not have an effect size from a prior study to use as a basis for conducting a power analysis. We decided on a sample size of 400, which would give us 80% power to detect a Cohen's *d* = 0.28 with a = 0.05 (two-tailed). A survey seeking 400 US residents was posted on Cloud Research using the same criteria as in Study 2. Participants were only allowed to proceed to the survey if they correctly answered four multiple-choice attention-check questions. In response, 402 participants completed the study. As in Study 2, we included 394 participants who indicated that they were born in the US (203 women, 187 men, 3 other gender, 1 unreported; mean age 42.44 years, *SD* = 12.39). Overall, 197 participants were randomly assigned to the PWE, and 197 to the Anti-PWE condition.

We used a biased questionnaire manipulation^58^ to manipulate the salience of PWE. Participants in the PWE condition were asked to respond to an adapted ten-item scale^41^. We adapted the items to increase the chances that participants would agree with them (e.g., "Most people who are willing and able to work hard have a good chance of succeeding;" α = 0.79). In the Anti-PWE condition, we took the ten items and reframed them to indicate the opposite idea (e.g., "Even people who are willing and able to work hard often don't get a chance to succeed;" α = 0.87; see Supplemental Material for detailed stimuli). Following previous research^59^, we presented participants with a biased response scale that had one *disagree* option (i.e., do not agree) and six *agree* options (i.e., agree slightly, agree somewhat, agree moderately, agree quite a bit, agree strongly, agree extremely). Next, we administered the measure of disturbance with income equality used in Study 2 (α = 0.96).

## Results

As in Study 2, the dependent variable indicated was highly right-skewed (*M* = 75.08, *SD* = 28.79, skewness = − 1.32, kurtosis = 3.63; see Fig. S3 in Supplementary Materials for the histogram), and non-normally distributed (skewness-kurtosis test c^2^(*df* = 2) = 60.256, *p* < 0.001. We thus analyzed the data using the nonparametric Wilcoxon Mann–Whitney rank-sum test. We found that participants in the anti-PWE condition were significantly more disturbed by the information about income inequality (rank sum = 41,400.50, expected = 38,707.50) than participants in the PWE condition (rank sum = 36,414.50, expected = 38,707.50), *z* = 2.12, *p* = 0.027.

Study 3 thus provided causal evidence for the idea that PWE leads people to accept income inequality. Participants exposed to the idea that hard work leads to success were less disturbed by information about factual information about high degrees of income inequality in society than participants exposed to the idea that hard work does not always lead to success.

### Study 4: Replication and extension

Although Studies 2 and 3 confirmed the relationship between PWE and support for inequality, as suggested by the machine learning model, there is quite a bit of conceptual overlap among the top predictors identified by the machine learning model, PWE, religiosity, and political orientation, which have been studied in the system justification literature^60^. Research has found that political orientation, right wing authoritarianism, social dominance orientation, and other constructs related to PWE are associated with people’s attitudes about inequality^61^. Thus, it is not clear whether the effect observed in Study 3 is actually due to PWE or because of other related constructs that might have been activated by our experimental manipulation. The goal of this study to test whether the findings from our Study 3 replicate even after we control for related constructs, such as system justification, belief in a just world, social dominance orientation, and right-wing authoritarianism.

## Method

We decided on a sample size of 200, which would give us 80% power to detect a medium effect, Cohen's *d* = 0.40, with a = 0.05 (two-tailed). A survey seeking 200 US residents was posted on Cloud Research using the same criteria as in Study 3. In response, 200 participants completed the study (80 women, 119 men, 1 unreported; mean age 43.39 years, *SD* = 12.75). Of these, 100 were assigned to the PWE condition and 100 to the anti-PWE condition. As in the previous studies, we excluded six participants who were not born in the US.

We used the same biased questionnaire manipulation as in Study 3. After the manipulation, we measured participants’ support for inequality (α = 0.96), along with a number of related constructs in randomized order: general system justification^62^ (α = 0.90), economic system justification^63^ (α = 0.92), general belief in a just world^64^ (α = 0.96), personal belief in a just world^64^ (α = 0.96), social dominance orientation^65^ (α = 0.95), and right-wing authoritarianism^66^ (α = 0.92). Finally, we measured participants’ political orientation (α = 0.97). Participants were asked to respond to all measures on 7-point scales (please see the survey document uploaded on OSF for details).

## Results

We tested whether the experimental manipulation predicted support for inequality above and beyond the seven additional predictors. As the other predictors were intercorrelated (*r*’s = 22–0.79), we ran a LASSO regression to sort out relevant from irrelevant predictors of participants’ support for inequality. Specifically, we included the experimental condition (PWE = 1; Anti-PWE = 0) and the seven related constructs as predictors. We selected the LASSO free parameter L value using tenfold cross-validation. The LASSO regression identified the experimental condition, general system justification, economic system justification, social dominance orientation, and personal belief in just world as relevant predictors. We next ran a linear regression including these predictors (see Table 3), which found a significant effect of experimental condition (*p* = 0.026) even after controlling for other relevant predictors.

**Table 3:** Regression results (Study 3).

Predictor	Beta coefficient	95% CI (lower bound)	95% CI (upper bound)	Standard error	*t(*df = 188)	*p*
Condition	7.59	0.91	14.28	3.39	2.24	0.026
Economic system justification	− 6.99	**− **12.22	**− **1.75	2.66	**− **2.63	0.009
General system justification	**− **6.27	**− **10.22	**− **2.32	2.00	**− **3.13	0.002
Social dominance orientation	**− **6.35	**− **10.54	**− **2.16	2.12	**− **2.99	0.003
Personal belief in just world	3.12	**− **0.20	6.43	1.68	1.86	0.065

Study 4 indicated that although past research has found that PWE is related to a number of other constructs, experimentally highlighting PWE increased people’s support for inequality even after controlling for related constructs. Thus, PWE influences support for inequality independent of other constructs.

### General discussion

The current research used a deep learning model to uncover the most important predictors of people's support for income inequality from a wide range of potential predictors assessed in the WVS. The model could accurately predict respondents ' views about income inequality based on WVS respondent's attitudes, values, and beliefs. A feature importance analysis identified three important classes of predictors: political orientation and associated beliefs, religiosity, and the importance of hard work (which is a core component of the Protestant work ethic). A correlational study verified that the more people endorse PWE, the less concerned they are about high degrees of income inequality in society. An experiment found that people exposed to the idea that hard work is important were less concerned about income inequality than those exposed to the idea that hard work is not all that important. A follow-up experiment confirmed that this experimental manipulation influenced the extent to which participants endorse PWE even after controlling for other related constructs, including system justification, belief in a just world, and social dominance orientation.

The current research complements recent research that has used machine learning methods to generate cause-effect hypotheses in psychology^31^. The present research counters the idea that machine learning emphasizes prediction at the cost of explanation^67^. That is, our deep learning model predicted participants' attitudes about income inequality with a Spearman correlation coefficient of 0.46, which has recently been categorized as very high^68^. Further, the feature importance analysis allowed us to explain respondents' attitudes about income inequality. Thus, given the range of current tools available, it is no longer the case that machine learning models are “black boxes”^69^.

A key methodological contribution of the current research is in documenting that researchers do not need to build deep neural networks from scratch if their goal is to generate hypotheses. If there are pre-existing neural networks built on the same dataset, then researchers can merely retrain the model to predict their outcome variable of interest without changing any model parameters; the retraining process would adjust all the weights in the model that contribute to the prediction. The current research demonstrates that this procedure works even if we change the outcome variable from a binary variable (as in the previous model that we reused^31^) to a continuous variable (as in our Study 1); we just needed to change the loss function of the model *binary cross-entropy loss* to *mean square error*. This procedure reduced the model building time by many folds. The current research thus suggests that if researchers wish to identify predictors of other variables included in the WVS, they can use the pre-existing model as a starting point. Notably, this procedure would be appropriate if researchers’ primary goal is to identify top predictors of outcome variables of interest. If their goal is to predict the outcome variable as accurately as possible, then they can use pre-existing models as a baseline but adjust all parameters to maximize accuracy.

This research contributes to the psychological literature on predictors of people’s acceptance of inequality. This work has identified predictors such as political orientation^19^, system justification^60^, belief about income mobility^21^, the salience of choice^22^, and so on^13^. Although PWE is related to a number of these constructs, including conservatism^45^, we found that PWE predicted people’s concern with inequality above and beyond their political orientation and other related constructs, such as system justification, belief in a just world, social dominance orientation, and right-wing authoritarianism. Many people likely assume that income inequality results from rich people's hard work and poor people's lack of hard work. Thus, the more they emphasize hard work, the more they accept inequality as justified. However, these people might be ignoring the contributions of economic, sociological, and cultural factors that contribute substantially to people's income. These include the education, occupation, and wealth of their parents^70^, the neighborhood in which they grew up^71^, the quality of the schools that they attended^72,73^, and the historical status of the ethnic and cultural groups to which they belong^74^.

This research contributes to the psychological literature on Protestant Work Ethic. This work has examined the implications of PWE for people's prejudice toward the poor^56^ and ethnic minorities^41^. We contribute to this literature by documenting the implications of PWE for people's attitudes about income inequality. Income inequality depends not only on the outcomes of low-status groups in society but also on the middle-status and high-status groups. For example, people may be unsympathetic toward the disadvantaged minorities but still oppose high degrees of inequality between the rich and the middle-class, or a high concentration of wealth in the very top end of the income distribution. The current findings suggest that PWE is relevant not just to people's attitudes about low-status groups, but also inequalities across groups. More broadly, the machine learning results suggest that PWE is among the top three classes of correlates of inequality acceptance (political orientation and religiosity are the other two). These findings suggest that PWE might be relevant to a broader range of outcomes than currently conceptualized in the psychological literature.

Although machine learning models serve as excellent tools for generating hypotheses in a theory-blind manner, they have several limitations. First, the hypotheses that can potentially be generated by machine learning models are restricted to the set of variables that are included in the dataset. If important predictors of the outcome variable are not included in the dataset, then the model cannot identify them as top predictors. Second, all machine learning models are approximate solutions, so no model can be proven to be the best possible model^75^—it is always possible that a better model exists, which means that the top predictors identified by the current model might change if a superior model is identified. In practice though, as long as researchers have identified a good enough model with reasonably high accuracy, a more accurate model would likely feature many of the same top predictors; however, the rank ordering of the top predictors is likely to shift. Finally, an important limitation of our research is that although the machine learning model identified the emphasis on hard work as a culture-general predictor of people’s attitudes about income inequality, we tested this relationship only in the US (given easy access to US participants). It would be important for future research to assess whether this finding replicates in other cultures sampled in the WVS.

## Supplementary Information

Supplementary Information.

## Data Availability

The survey data and all relevant files are available at https://osf.io/q5xzy/.
