# Supplementary material for: A deep learning model identifies emphasis on hard work as an important predictor of income inequality
Source: Sci Rep. 2022 Jun 14;12:9845. doi: 10.1038/s41598-022-13902-x (PMC9194778; doi:10.1038/s41598-022-13902-x)
Supplement: Supplementary file 1 — Supplementary Information. [file 41598_2022_13902_MOESM1_ESM.docx]

**A deep learning model identifies the emphasis on hard work as an important predictor of income inequality**

Abhishek Sheetal^1,2^, Srinwanti H. Chaudhury^3^, & Krishna Savani^2,4*^

^1^School of Business and Law, Central Queensland University, Rockhampton, Australia

^2^Faculty of Business, The Hong Kong Polytechnic University, Kowloon, Hong Kong

^3^Business School, University of Queensland, Brisbane, Australia

^4^Nanyang Business School, Nanyang Technological University, Singapore, Singapore

^*^krishna.savani@polyu.edu.hk

**Supplementary Materials**

May 25, 2022

**Study 1: Partial Dependence Plots**

**Figure S1**. Relationship between the top 16 predictors (standardized to range from 0 to 1; X-axis) according to the feature importance analysis, and the dependent measure (i.e., the extent to which people believe that income inequality is necessary as an incentive; Y-axis). Note that the depicted range of the Y-axis varies across different predictor variables.


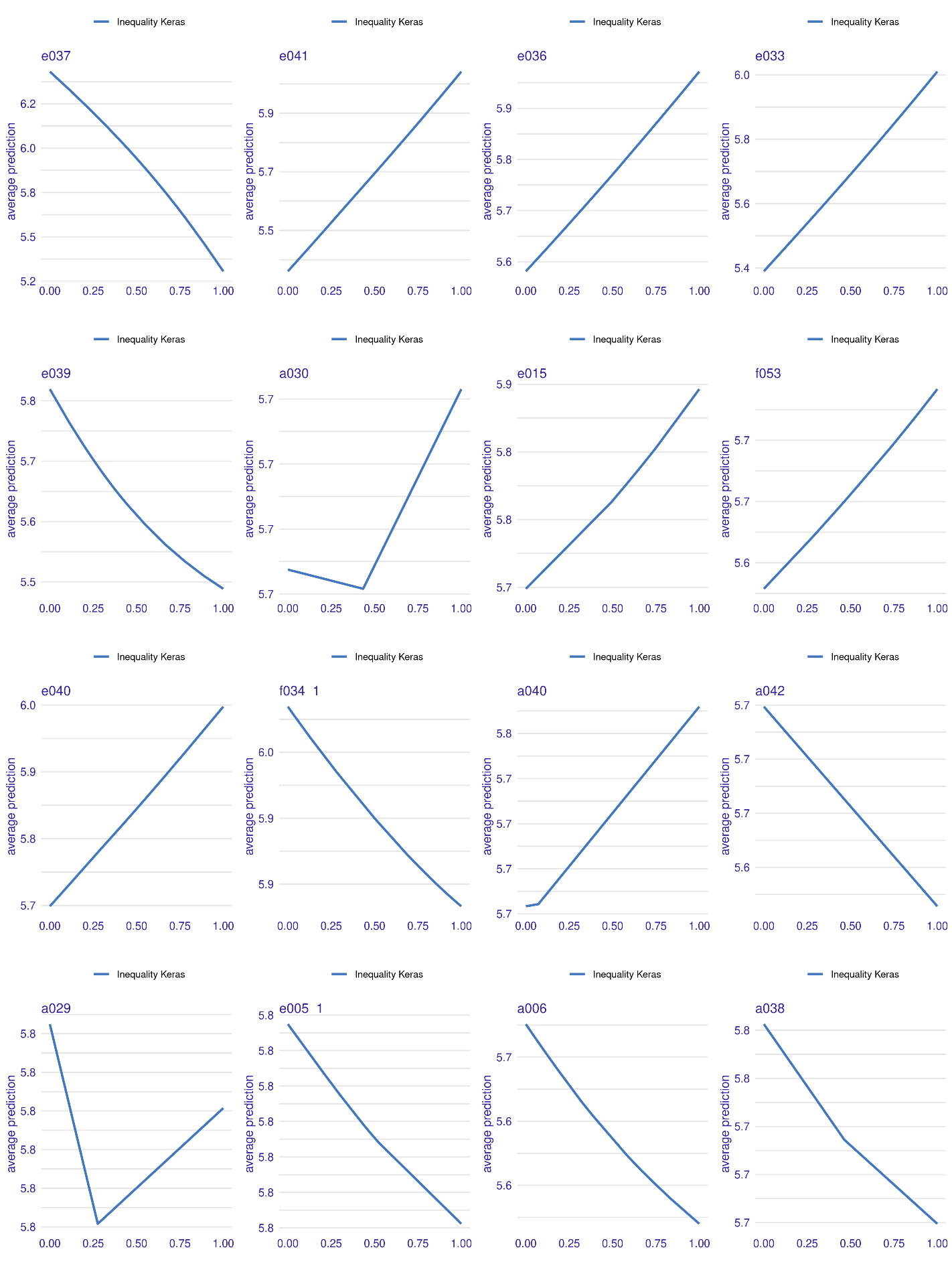


Information about machine learning model runtime:

Closed form OLS methods have a computational complexity of O(C^2^N) (Huang & Pan, 1998). Here, O refers to order of complexity, C refers to the number of predictors, and N refers to the number of observations. In other words, the time taken to solve an OLS problem is proportional to the square of the number of predictors multiplied by the number of observations. Thus, OLS has linear time complexity in terms of number of observations. This might change if C >> N but for most practical applications, C << N.

All machine learning models, however, have a polynomial run time complexity O(N^m^), where m > 1 (Kearns, 1990). In practice, polynomial time complexity algorithms are never executed in any binary digital computer without attaching run time limits. Such models could potentially keep running for years. Researchers working with polynomial time complexity algorithms define acceptable model convergence; when this is achieved, the model stops running. To estimate estimated completion time, researchers need to check the epoch run time. For example, if one epoch takes 10 seconds to run, and if we set a maximum of 100 epochs per model, and set 1000 hyperparamater search runs, then the model training would take 10x100x1000 seconds, or approximately 12 days. The epoch time depends on the power of the computer that the model is run on.

In the current research, as we reused an existing deep learning model and did not conduct a fresh hyperparameter search; however, we ran the model for 200 epochs. The compute time was approximately 60 seconds X 200 epochs = 200 minutes. This analysis was run using Intel’s PlaidML libraries to conduct lower-level matrix multiplications on an AMD WX5100 graphics card with OpenCL drivers.

**Study 2: Correlational Study**

**Dependent Measure**

In this task, you will read about some factually correct statistics about income and wealth in the US. Please indicate your reactions to the statistics.

1. Recent statistics show that 3 richest men in the US own more wealth than the bottom 50% of Americans combined.

2. Recent statistics show that the top 10% of Americans earn 9 times more income per year than the bottom 90% *combined*.

3. Recent statistics show that in 1960, the average CEO earned 50 times as much as the average worker. In 2019, the average CEO earned 300 times as much as the average worker.

4. Recent statistics show that between 1990 and 2019, the average worker’s salary has risen by less than 5%, whereas the average CEO’s salary has risen by 500%.

5. Recent statistics show that the U.S. ranks 93th out of 133 countries in the world in terms of income inequality, in the immediate company of Cameroon (a very poor West African country) and Uruguay (a poor South American country). All European countries and Canada rank well above the U.S. in terms of income inequality.

For each of the above items, participants were asked:

How disturbed are you by this finding?

(Slider scale ranging from 0: *Not very disturbed* to 100: *Extremely disturbed*)

**Figure S2.** Histogram of the dependent variable.

**Study 3: Experiment**

Items used in the *Protestant work ethic* condition (adapted from Katz & Hass, 1988):

1. Many people spend too much time in unprofitable amusements.
2. Our society would have fewer problems if people had less leisure time.
3. Money acquired easily is usually spent unwisely.
4. Many people who don't succeed in life are just plain lazy.
5. Most people who are willing and able to work hard have a good chance of succeeding.
6. People who fail at a job have usually not tried hard enough.
7. Life would have very little meaning if we never had to suffer.
8. The person who can approach an unpleasant task with enthusiasm is the person who gets ahead.
9. If people work hard enough, they are likely to make a good life for themselves.
10. I feel uneasy when there is little work for me to do.

**Items used in the Anti-Protestant work ethic condition** (adapted from Katz & Hass, 1988)**:**

1. Many people don’t have enough time for amusements.
2. Our society would have fewer problems if people had more leisure time.
3. Money acquired easily is usually saved.
4. Many people who don't succeed in life are just plain unlucky.
5. Even people who are willing and able to work hard often don’t get a chance to succeed.
6. Even people who have tried very hard sometimes fail at a job.
7. Life would be much more enjoyable if we never had to suffer.
8. Even people who approach an unpleasant task with enthusiasm often don’t get ahead.
9. Even people who work very hard sometimes cannot make a good life for themselves.
10. At times I long for a time when there is little work for me to do.

**Figure S3.** Histogram of average income inequality scores (for those born in US)

**References**

Huang, X., & Pan, V. Y. (1998). Fast rectangular matrix multiplication and applications. *Journal of Complexity*, *14*(2), 257-299.

Katz, I., & Hass, R. G. (1988). Racial ambivalence and American value conflict: Correlational and priming studies of dual cognitive structures. *Journal of Personality and Social Psychology*, *55*(6), 893-905.

Kearns, M. J. (1990). *The computational complexity of machine learning*. MIT press.
